# Entirely Carbohydrate-Based Vaccines: An Emerging Field for Specific and Selective Immune Responses

**DOI:** 10.3390/vaccines4020019

**Published:** 2016-05-20

**Authors:** Sharmeen Nishat, Peter R. Andreana

**Affiliations:** Department of Chemistry and Biochemistry, School of Green Chemistry and Engineering, University of Toledo, Toledo, OH 43606, USA; Sharmeen.Nishat@utoledo.edu

**Keywords:** *T-cell dependent* immune response, zwitterionic polysaccharides, carbohydrate-based vaccines, PS A1

## Abstract

Carbohydrates are regarded as promising targets for vaccine development against infectious disease because cell surface glycans on many infectious agents are attributed to playing an important role in pathogenesis. In addition, oncogenic transformation of normal cells, in many cases, is associated with aberrant glycosylation of the cell surface glycan generating tumor associated carbohydrate antigens (TACAs). Technological advances in glycobiology have added a new dimension to immunotherapy when considering carbohydrates as key targets in developing safe and effective vaccines to combat cancer, bacterial infections, viral infections, *etc*. Many consider effective vaccines induce *T-cell dependent* immunity with satisfactory levels of immunological memory that preclude recurrence. Unfortunately, carbohydrates alone are poorly immunogenic as they do not bind strongly to the MHCII complex and thus fail to elicit T-cell immunity. To increase immunogenicity, carbohydrates have been conjugated to carrier proteins, which sometimes can impede carbohydrate specific immunity as peptide-based immune responses can negate antibodies directed at the targeted carbohydrate antigens. To overcome many challenges in using carbohydrate-based vaccine design and development approaches targeting cancer and other diseases, zwitterionic polysaccharides (ZPSs), isolated from the capsule of commensal anaerobic bacteria, will be discussed as promising carriers of carbohydrate antigens to achieve desired immunological responses.

## 1. Introduction

**Figure 1:**
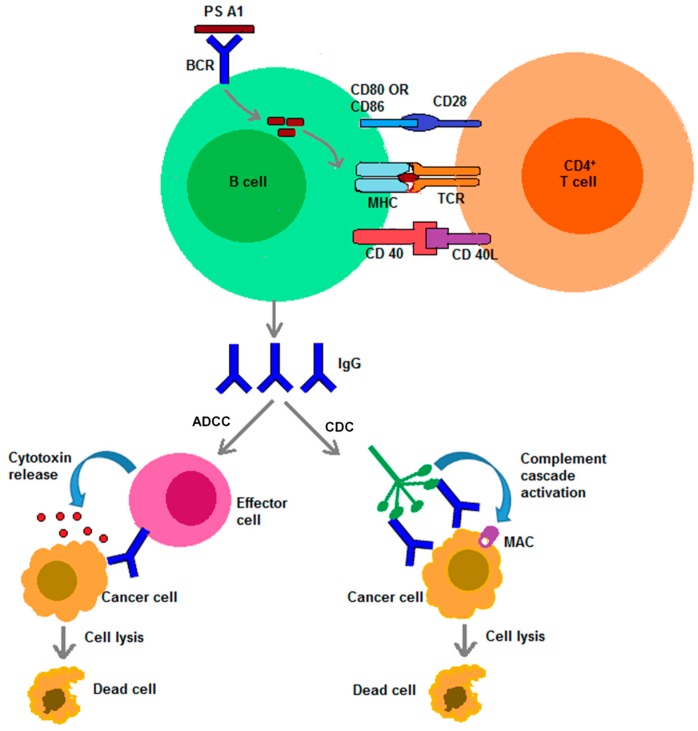
Cancer cell death through immune cytotoxicity (ADCC and CDC).

The idea of vaccine development commenced with the observation that malignant tumors could be treated by repeated inoculation of erysipelas [1], an acute infection caused by a beta-hemolytic group A *Streptococcus* bacteria. Numerous experimental approaches, based on that seminal observation brought noteworthy progress to the field of vaccinology, which has demonstrated that vaccines are potent in disease prevention. Vaccines typically protect individuals by empowering the human to induce humoral and/or cellular immunity against pathogens [2]. Humoral responses from antigens arise as a result of binding to the B-cell receptor to invoke B-lymphocytes to produce high avidity but low affinity antibody IgM. In order to get high affinity IgG antibodies, additional stimulation from activated T-helper cells is required for the proliferation and differentiation of naïve B-cells to antibody secreting plasma cells (Figure 1). To activate CD4+ T-helper cells, the antigens need to be processed in the antigen presenting cell (APC), bind with major histocompatibility complex II (MHCII) and then presented on the surface to the α,β-T-cell receptor of naïve T-lymphocytes [3]. To capitalize on this most effective immune response, aside from whole-cell traditional vaccine approaches (attenuated or dead microbes or components of microbes), many synthetic and recombinant vaccines are the subject of current and active research [4].

The vast majority of known pathogens have dense distributions of complex polysaccharides, oligosaccharides and glycans on their cell surface; known as the glycocalyx [5]. Aberrant glycosylations on the surface of cancer cells are known to exist as a direct result of down-regulated protein expression giving rise to tumor associated carbohydrate antigens (TACAs) [6,7]. Carbohydrates have long been known to elicit *T-cell independent immune responses* and have therefore failed in achieving isotype switching from IgM to IgG antibody and memory cell production (plasma cells), which make them poorly immunogenic [8,9]. To overcome this grand challenge, carbohydrates have been conjugated to immunogenic carrier proteins such as bovine serum albumin (BSA) [10], keyhole limpet hemocyanin (KLH) [11], diphtheria toxin mutant (CRM_197_) [12], tetanus toxoid (TT) [12], diphtheria toxoid (DT) [13], ovalbumin [14], human serum albumin (HSA) [15], meningococcal outer membrane protein complex (OMPC) [12], *Hemophilus influenzae* protein D [12], *Pseudomonas aeruginosa* exotoxin A (rEPA) [16] and others, so that a *T-cell dependent* immune response can be induced resulting in increased production of antibody titers, isotype switching from IgM to IgG *via* plasma cells and memory T- and B-cells [17]. However, carrier proteins, being self-immunogenic, can lead to increased peptide specific antibody production resulting in the suppression of immunity towards the targeted carbohydrate antigen(s) [18].

Alternatives to carrier proteins, for eliciting a *T-cell dependent* immune response, can potentially lead to enhanced immunogenic specificity towards carbohydrate antigens. Literature precedence revealed a subpopulation of T-lymphocytes, known as natural killer T-lymphocytes (NKTs), which can recognize glycolipids on the surface of CD1d (a non-classical MHC molecule). CD1d has a hydrophobic antigen binding pocket, therefore, the lipid portion binds in the hydrophobic pocket and the carbohydrate portion is exposed for T-cell recognition. NKT cells, upon recognizing carbohydrate antigens, can rapidly secrete a variety of cytokines such as IFN-γ, IL-4, TNF and activate other immune cells like dendritic cells, natural killer cells, B-cells, *etc*. [19]. This is how T-cell mediated immune responses towards carbohydrate antigens can be achieved in the absence of carrier proteins. Along these lines, zwitterionic polysaccharides (ZPSs), isolated from the capsule of commensal anaerobic bacteria are also known to elicit *T-cell dependent* immune responses [2,20]. ZPSs are able to recruit lymphocytes, neutrophils, and macrophages [21]. What is also known is that removing either the negative or positive charge, or both, make it non T-cell stimulatory [20]. Utilizing the idea of ZPSs as immunogens, vaccine constructs can be designed for carbohydrate antigen-ZPS conjugates so that *T-cell dependent* carbohydrate specific immunity can be induced in the absence of carrier proteins or other carriers *and* attain the important isotype switching event. To elaborate further, it is well-known that antibodies have the ability to eliminate cancer cells by complement-dependent cytotoxicity (CDC) and/or by antibody-dependent cellular cytotoxicity (ADCC) through NK cells and macrophages (Figure 1) [22]. This review is therefore intended to provide a detailed discussion about the immunogenicity of ZPSs and related vaccines, more specifically about how conjugate polysaccharide A1 (PS A1) is currently being utilized to combat cancer.

## 2. *T-cell Independent* Immune Responses

Carbohydrates have long been known to be *T-cell independent* and only elicit the innate immune response primarily through pathogen associated molecular pattern (PAMP) receptors [23] and weak adaptive immune responses *via* cross-linking with B-cell receptors. There are several well characterized pathways of adaptive immunity. Firstly, *thymus dependent* immunity, which relies on T-helper cells to coordinate an immune response *via* secretion of various cytokines and secondly, the *thymus independent* pathway, which is a B-cell dependent response against various antigens [24]. The *thymus independent* pathway I is generally reserved for lipopolysaccharides such as various endotoxins native to gram negative bacteria. The *thymus independent* pathway II is an immune pathway that is reserved for high repetition polysaccharides and only activates mature B-cells, *i.e.*, B-cells already programmed against a particular antigen. Generally, the repetitious polysaccharides lead to a secretion of high spectrum, low specificity IgM antibodies.

High repetition polysaccharide antigen units are recognized by antigen presenting cells by a class of molecules called pathogen recognition receptors (PRRs) [25]. They are endocytosed, cleaved into smaller subunits and presented on the surface of antigen presenting cells for B-cell recognition. The APC, in this pathway, presents an abundance of the same high repetition carbohydrate to the B-cell, causing the surface antibodies (B-cell receptors; BCRs) to crosslink and begin production of IgM antibodies. Unfortunately, there is no memory component to this immune pathway [8,9]. However, enhancement of glycan immunogenicity by covalent conjugation to a protein scaffold was explored in 1931 by the seminal work of Avery and Goebel [26]. Taking advantage of this finding, a number of polysaccharide-protein conjugate vaccines were synthesized [27,28,29] and promising immunological responses including long lasting immunity concomitant with polysaccharide specific immunoglobulin class switching (IgM to IgG) was attained. T-cell mediated immune responses from protein conjugates initiates with antigen uptake followed by proteolytic cleavage to process the antigen which is ultimately presented on the MHCII complex to the T-cell receptor. With the aid of a T-cell signal, high affinity antibodies and cellular memory responses can be generated that target the carbohydrate antigen.

## 3. Entirely Carbohydrate Vaccine Conjugates

Although in 1923, Heidelberger and co-workers had shown that pneumococci capsular polysaccharides could induce immunity [30], up until the 80’s carbohydrates were not extensively used in vaccinology against disease-causing pathogens. However, due to the lack of long-lasting immune protection by polysaccharide antigens, very few polysaccharide vaccines are currently commercially available. Those that are include the Vi polysaccharide vaccine [31,32] and *Neisseria meningitides* polysaccharide vaccine [33]. On the other hand numerous protein conjugated bacterial capsular polysaccharide vaccines are commercially available including Hiberix^®^ (Glaxo SmithKline, Rixensart, Belgium) (first polysaccharide conjugated vaccine), Prevenar 13^®^ (Wyeth Pharmaceuticals Inc., Pearl River, NY, USA ), Menjugate^®^ (Chiron, Emeryville, California, USA), PedvaxHIB^®^ (Merck, Kenilworth, NJ, USA)and others [12]. These conjugates are known to confer effective, long lasting immune protection, which is indicative of the importance of carrier proteins in improving the immune response of poorly immunogenic polysaccharides.

### 3.1. Vi Polysaccharide Vaccine

Vi is a capsular polysaccharide present in Salmonella typhi, a typhoid causing pathogen in humans [34]. Vi is composed of α-(1,4)-linked 2-acetamido-2-deoxy-d-galacturonic acid with variable O-acetylation at C-3. It is reported to interact with toll-like receptor (TLR)-2 /TLR-1 [35], can induce expression of co-stimulatory molecules [36] on the monocyte and secret pro-inflammatory cytokines. Vi immunization results mostly in the production of IgM antibodies with some isotype switching to IgG3 [37]. Similar antibody responses obtained from Vi immunized αβ-T-cells or γδ-T-cells deficient mice is suggestive of a T-cell independent response. This is reported to be induced upon MyD88 (TLR adaptor) dependent stimulation [38].

Despite the preponderance for a T-cell independent immune response, the non-conjugated Vi vaccine is licensed. It can infer protection for approximately three years [39], but nominally it has failed to protect children under 2 years of age. To get long lasting immune protection, Vi is coupled to a recombinant mutant of Pseudomonas aeruginosa exotoxin A (Vi-rEPA) [40] or a carrier protein [41]. In a report, unconjugated Vi polysaccharide was noted to promote memory B- and T-cells when mucosal adjuvant AFCo1 or AFPL1 was used during immunization through the nasal route [42]. This observation suggested that with the aid of proper adjuvants, immunogenicity can be improved even after by-passing protein conjugation.

### 3.2. Neisseria Meningitides Polysaccharide Vaccine

Meningitis is a fatal disease caused by the gram negative bacteria Neisseria meningitides. The bacterial capsule consists of an α(2,8)-linked polysialic acid homopolymer which has antigenicity similar to human fetal neural cell adhesion molecules [43]. This antigenic similarity can trigger immunogenic tolerance which can render the polysaccharide vaccine very poorly immunogenic. However, as this capsular polysaccharide (CP) is a major virulence factor [44,45], the purified CP vaccine was developed against different serotypes of this bacteria [46,47]. The unconjugated capsular polysaccharide-based vaccine Menomune^®^ (Sanofi Pasteur, Pocono, PA, USA), which is useful for serotypes A/C/Y/W-135 can also induce short-term protection of approximately 2–3 years [33]. To achieve long-term protection, meningococcal groups A, C, Y and W-135 capsular polysaccharides are individually conjugated with either diphtheria toxoid or CRM_197_ to achieve quadrivalent conjugate vaccines which are licensed as Menactra^TM^ (Sanofi Pasteur) [48] or Menveo^®^ (Novartis Vaccines & Diagnostics s.r.I., Siena, Italy) [49] respectively.

### 3.3. Tumor Associated Carbohydrate Vaccine

Tumor associated cell surface glycans are often considered as “self-antigens” to immune surveilance, therefore, triggering immune-based elimination of cancer cells has been a growing area of research for many years. In considering these “auto-antigens”, cancer vaccine development must rely on breaking this immune tolerance towards the antigen expressed on tumor cells. Conjugation of TACAs to carrier proteins [50], nano-particles [51], virus-like particles [52], lipids [53], *etc.*, can make these antigens “look” more foreign-like, hence, these approaches are currently being explored to overcome immune tolerance and effectively provoke long-lasting immune responses towards cancer carbohydrate antigens. Unfortunately, neither carbohydrate-based or protein-carbohydrate conjugate vaccines are commercially available. Some protein-carbohydrate antigen conjugate vaccines [(Sialyl Thomsen-nouveau (STn), Ganglioside GM2, Thomsen-Friedenreich (TF)], while have been studied in clinical trials, have failed to exhibit expected therapeutic effects [54,55,56,57,58,59]. Therefore, more intense research coupled with alternative strategies are required to identify the proper carrier, adjuvant system or delivery method to attain effective cancer vaccines with therapeutic efficacy. Having these sugars as part of the tumor cell’s glycocalyx (cell surface carbohydrates) makes this a viable undertaking because, collectviely, they represent the first line of cellular defense.

## 4. Zwitterionic Polysaccharides: A Means to T-cell Activation

**Figure 2:**
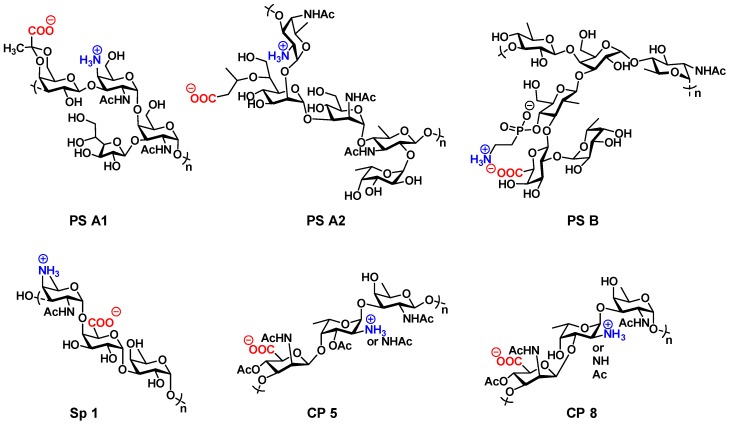
Structures of zwitterionic polysaccharides (ZPSs).

Zwitterionic polysaccharides (ZPSs), comprising both positive and negative charges on adjacent monosaccharide units, are known as being T-cell stimulatory independent of peptides, proteins or lipids. They are also known to be processed by antigen presenting cells (APCs) and presented as MHCII-ZPS complexes on the surface for α/β-TCR recognition of CD4+ T-cells that can promote immunoglobulin class switching from IgM to IgG [60]. The immunomodulatory property of ZPSs can efficiently replace the practice of carrier protein conjugation to polysaccharides or carbohydrate oligosaccharides and holds promise to provide a unique solution to assembling entirely carbohydrate-based vaccines; a means of attaining carbohydrate homogeneity to generate specific immune responses towards carbohydrate antigens which otherwise could be suppressed by immunogenic carrier proteins [18]. The majority of naturally occurring polysaccharides are either neutral (Group A Streptococcal polysaccharide) [61] or anionic (Vi polysaccharide) [31,32] and while these moieties can be processed in APCs, they ultimately fail to bind with the MHCII complex most likely due to the absence of electrostatic interactions and as a result generate *T-cell independent* immunity [62]. However, very few zwitterionic polysaccharides, on the capsules of certain commensal or pathogenic bacteria, have been explored in the realm of vaccinology as a means to provoke *T-cell dependent* immune responses. However, there are some that have been employed to elicit a *T-cell dependent* immune response including polysaccharide A1 (PS A1) and PS B from *Bacteroides fragilis* (ATCC 25285/NCTC 9343) [63], PS A2 from *Bacteroides fragilis* 638R [64], specific type 1 polysaccharide (Sp1) from *Streptococcus pneumoniae* [20,65], and CP5/CP8 from *Staphylococcus aureus* [66] (Figure 2). In addition, chemically synthesized ZPSs are also known to augment immunogenicity [67]. The detailed mechanistic studies with ZPSs, especially PS A1, demonstrates the resemblance of ZPSs to carrier proteins and includes pathways that lead to both innate and adaptive immune responses [68].

### 4.1. Zwitterionic Capsular Polysaccharides from B. fragilis (PS A1, PS B, PS A2)

*Bacteroides fragilis* is a gram negative anaerobic commensal bacterium which is the most frequently isolated species from clinical intra-abdominal abscesses [69]. An experimental induction of abscess in a rat model by intra peritoneal implantation of capsular polysaccharide complex (CPC) from *B. fragilis* with sterile cecal content adjuvant revealed this CPC was a virulence factor [70]. Therefore this CPC can be utilized as an antigenic target. This was further validated by immunizing a rat model with CPC after *B. fragilis* challenge; CPC immunization protected rats from abscess formation [71,72]. In another study, transfer of splenocytes from *B. fragilis* CPC immunized mice to non-immunized mice protected recipients from abscess formation [73]. This observation, using the mouse model, is a direct indication of cellular immunity generated by CPC [73].

Among eight different polysaccharides isolated from *B. fragilis* (not all zwitterionic), ZPS PS A1 is the most abundant followed by ZPS PS B. Both of these capsular polysaccharides contain a zwitterionic charge character that is necessary for immunological T-cell mediated responses [20,74]. PS A1 consists of a tetrasaccharide core repeating unit (~120 repeating units) with a molecular weight of ~110 kDa having alternate opposite charges on adjacent monosaccharide units. The repeating unit is comprised of 2,4-dideoxy-4-amino-d-*N*-acetylfucose, d-*N*-acetylgalactosamine, d-galactopyranose, and d-galactofuranose with a 4,6-pyruvate acetal attached to the galactopyranose [74]. PS B, another high molecular weight capsular zwitterionic polysaccharide from *B. fragilis* is comprosed of repeating sugars [→3)-β-d-Qui*p*NAc (1→4), α-d→Gal*p* (1→4), α-l-QuiNAc (1→3), and branched from 3′-galactose is β-d-Glc*p*NAc (1→3), α-d-Gal*p*A(1→3), and α-l-Fuc*p*(1→2) [74]. PS A2 is a zwitterionic polysaccharide, isolated from the *B fragilis* 638R strain and is comprised of a pentasaccharide core repeating unit with the sequence: [→3)-α-d-AAT*p*-(1→2)-α-d-Hep*p*NAc-(1→3)-α-d-Man*p*NAc-(1→4)[α-l-Fuc*p*-(1→2)]-β-d-ADG*p*A-(1→] [64].

It has been observed that removal of zwitterionic charge from PS A1 makes it non-immunogenic [20] which reflects the importance of the zwitterionic character for immune stimulation. Detailed mechanistic studies have shown that processing and presentation of PS A1, in an MHCII complex, is not the exact same but similar to conventional protein antigen processing mechanism(s). PS A1 is now known to be internalized by APCs through pinocytosis or receptor mediated endocytosis [75]. In the endosomal compartment, PS A1 undergoes fragmentation *via* a nitric oxide mediated oxidative burst pathway resulting in degradation of PS A1 into lower molecular weight units [76]. After endosomal maturation, it is fused with the lysosome to form MHCII compartments where PS A1 colocalizes with both lysosomal associated membrane proteine-1 (LAMP-1) and HLA-DM. HLA-DM catalyzes the binding of PS A1 to the MHCII complex. Approximately 12-15 kDa PS A1 fragments are required for effective antigen presentation [77]. After being loaded onto the MHCII molecule, as the important MHCII – PS A1 complex, the PS A1 is then presented to the αβ-TCR of CD4+ T-cells. PS A1 can also cause the induction of costimulatory molecules CD40 and CD86 or CD80 on the APC surface [78].

**Figure 3:**
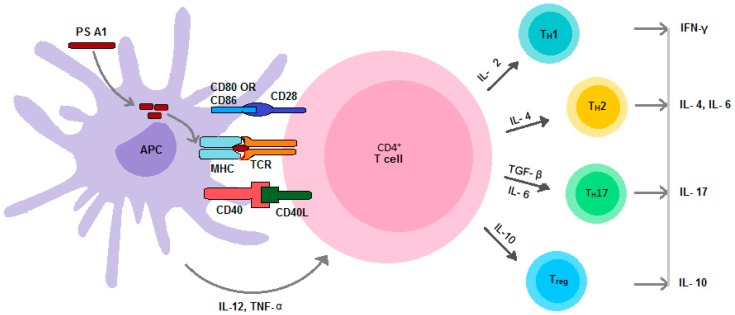
Activation and differentiation of CD4+ T-cells.

PS A1 is also known to bind to toll like receptor-2 (TLR-2) [68] which is primarily responsible for invoking innate immune responses. Binding of PS A1 to TLR-2 activates dendritic cells to release IL-12, which activates signal transducer and activator of transcription 4 (STAT 4) of CD4+ T-cells to stimulate T-cell differentiation into T_H_1 cells and release IFN-γ. Therefore, TLR-2 can mediate the inflammatory response and synchronize innate and adaptive immunity [79]. PS A1 can also stimulate T-helper cells to differentiate into T_H_2 upon induction of the IL-4 cytokine. IL-6 and TGF-β mediated differentiation of the T-helper cell to T_H_17 can be evaluated by the production of IL-17A [80]. Experimental evidence has also demonstrated that ZPSs can induce regulatory T-cells (T_reg_) to release pro-inflammatory cytokine IL-10 [81] that can protect from inflammation [82]. The aforementioned phenomena of helper T-cell differentiation (Figure 3) was evaluated experimentally by monitoring cytokine profiles in the anti-sera of immunized mice or by using transgenic mice [2,83].

T-helper cell polarization can play a major role in generating long-lived immunoglobulin G (IgG). The overall humoral responses would result in both IgM and IgG production. ZPSs, when cross-linked with B-cell receptors (BCRs), can invoke a short-lived IgM response much like non-ZPSs. In addition, ZPSs can also induce memory B-cell production and isotype switching with the aid of activated T-cells in analogy to how a protein antigen does. This process initiates when ZPSs, expressed as an MHCII-ZPS complex on the surface of B-cells, encounter ZPS primed T-cells and get necessary costimulation (Figure 3). The B-cells are then transformed into long-living plasma cells that can generate immunoglobulin from isotype switching and memory B-cells [2].

### 4.2. Zwitterionic Capsular Polysaccharide Sp 1 from S. pneumoniae

Sp1 is a zwitterionic polysaccharide present in the capsule of the gram positive bacterium *Streptococcus pneumoniae* and is comprised of a trisaccharide repeating unit carrying an alternating single positive charge and two negative charges. The repeating unit is: [→3-α-2,4-dideoxy-4-amino-d-FucNAc-(1→4)-α-d-GalA*p*-(1→3)-α-d-GalA*p*-(1→] [84]. Due to the presence of the electrostatic charge, Sp1 can promote a *T-cell dependent* immune response in a way similar to PS A1, including internalization through the APC, nitric oxide dependent oxidative processing to low molecular weight fragments [85] and presentation on the MHCII complex to T-cell receptors.

### 4.3. Zwitterionic CP5 and CP8 from S. aureus

Among the variety of clinical isolates of *S. aureus* strains, serotype 5 and 8 containing capsular polysaccharide 5 (CP5) and 8 (CP8) respectively are the most prevalent [86,87]. CP5 and CP8 are very similar in structure, both comprising *N*-acetyl mannosaminuronic acid, *N*-acetyl-l-fucosamine and *N*-acetyl-d-fucosamine as integral parts of trisaccharide repeating units with varying sites of acetylation and different linkages between sugars [88]. The zwitterionic character arises due to the positively charged amine (partially acetylated) on a fucose ring and the negatively charged carboxylic group on *N*-acetyl mannosaminuronic acid [66].

## 5. Zwitterionic Polysaccharides in the Development of Cancer Vaccines

### 5.1. PS A1

To utilize the inherent immunogenicity of PS A1, our group was the first to report on the synthesis and immunological evaluation of an entirely carbohydrate-based vaccine construct [89]. Due to aberrant glycosylation on the surface of cancer cells, a number of TACAs have been identified including Tn (Thomsen-nouveau), STn (Sialyl-Tn), TF (Thomsen-Friedenreich), Globo H, GM2, GM3, *etc*. [90]. Our approach in using PS A1 was to first synthesize aminooxy TACA derivatives and then conjugate them to chemically oxidized PS A1, affording anti-cancer vaccine constructs [89,91].

Site-specific chemical modification of PS A1 was conducted by oxidizing vicinal diols of the galactofuranose ring with NaIO_4_ and the resultant aldehyde was subsequently condensed with aminooxy Tn (Thomsen-nouveau) antigen forming a physiologically stable oxime bond. Conjugate formation was confirmed by observing the presence of two oxime doublets (E/Z isomers) using NMR. A fluorescent labeling study allowed us to determine that 35% Tn antigen loading had occurred in the newly formed Tn-PS A1 construct [92]. After the noted conjugation, C57BL/6J mice were immunized with Tn-PS A1 in either the presence or absence of TiterMax^®^ Gold adjuvant (Sigma-Aldrich, St. Louis, MO, USA). A Tn specific, highly robust immune response (IgM and IgG, IgG3 ) was obtained which implied that chemical modification does not abrogate immunogenicity of PS A1. Since, similar antibody responses were obtained from adjuvant free vaccinated mice sera, PS A1 can be considered to play a dual role as both a carrier and adjuvant.

In a follow up report, anti-Tn-PS A1 sera containing the Tn specific IgG3 antibody was analyzed for the ability to recognize cancer cells using flow cytometry (FACS) [93] and it was revealed that the IgG3 antibody binds to Tn expressed on human tumor cell lines such as MCF 7, MDA-231, Jurkat (leukemia) and Panc-1 (pancreas). On the other hand, negligible Ab binding was observed with U251 (glioblastoma), human peripheral blood mononuclear cells and human bone marrow cells, which do not express the Tn antigen. The cytokine profile for both the anti-PS A1 sera and anti-Tn-PS A1 sera was analyzed so that a general understanding of the immune mechanism could be elucidated. A substantial amount of pro-inflammatory IL-2 (118 pg/mL) and anti/pro-inflammatory IL-6 (62 pg/mL) along with a small amount of anti-inflammatory IL-10 (16 pg/mL) and pro-inflammatory IL-17A (18 pg/mL) were observed when anti-PS A1 sera was used. Detection of a significant amount of IL-2 coincided exactly with the observation of Mazmanian *et al.* that *B. fragilis* colonization can correct splenic T_H_1/T_H_2 imbalance of germ free mice [78]. Production of IL-10 which signals to generate immune suppressive regulatory T-cells (T_regs_) is completely consistent with the role of PS A1 to prevent intestinal inflammatory disorder [94]. Surprisingly, a significant amount of IL-17A (58 pg/mL) was observed in anti-sera from Tn-PS A1 immunization. IL-17A is known to recruit neutrophils to induce inflammation [95] and is associated with the T_H_17 paradigm challenging T_H_1/T_H_2 immune response. Based on literature precedence [96,97], IL-17A can be considered to play a vital role in inducing anti-tumor effects including inducing potent tumor killing *via* T_H_17. Significant decrease in IL-2 (14 pg/mL), disappearance of IL-6, the presence of IL-4 (9 pg/mL) and a similar amount of IL-10 (12 pg/mL) gives rise to a distinctive immune response elicited by the Tn-PS A1 construct over that of PS A1. Excessive release of pro-inflammatory cytokines (termed as the cytokine storm) [98] can result in significant damage to body tissues, even cause death by blocking off the respiratory path. Therefore, considering this phenomena in conjunction with reports demonstrating the generation of immune suppressive T_regs_ restrains pro-inflammatory cells (T_H_1 or T_H_17) [99], it is reasonable to argue that the Tn-PS A1 vaccine elicits a balanced *T-cell dependent* immune response that can abrogate uncontrolled inflammation, yet potentially generate anti-tumor immune responses at optimum levels.

A circular dichroism (CD) spectrum of the PS A1 helical structure was also reported to having a high degree of alpha helical content [100]. Our group reported the CD spectra [92] of Tn-PS A1 at 3.6 ≤ pH ≤ 8.4, illustrating a decrease in alpha-helical character compared to native PS A1. Complete loss of alpha-helicity was observed at 8.5 ≤ pH ≤ 3.5. This observation implies that alpha-helical character is not the critical factor for immune activation, which is contrary to earlier reports [75] concerning the structural requirements of PS A1 for biological activity. As our reported NaIO_4_ method for chemical modification of PS A1 does not alter the zwitterionic character required for a robust immune response, it can be concluded that zwitterionic charge character does indeed play a vital role for *T-cell dependent* immune responses [20].

With the successful synthesis and analyses of immune responses from our Tn-PS A1 vaccine construct, our group was further inspired to synthesize a number of entirely carbohydrate-based immunogens employing our linker-free antigen synthesis strategy [89,101,102,103]. Immune suppression to the target antigen can occur when an immune response is directed towards a linker itself [104]. Therefore, as a continuation of our strategy, an aminooxy derivative of the TF antigen (found on cancerous mucin proteins and over-expressed on breast, prostate, colon, liver, stomach, bladder cancer cells) [105] was conjugated to PS A1 to access TF-PS A1 vaccine construct [91]. TF-PS A1 immunized anti-sera showed TF specific antibody responses. Mouse sera, containing TF specific polyclonal antibodies, efficiently recognize TF expressing cancer cells [106].

### 5.2. PS B

Along the same lines as our PS A1 approach, we were the first group to explore the immunogenicity of *B. fragilis* capsular polysaccharide PS B. To further support the idea that *T-cell dependent* immune responses were generated by zwitterionic polysaccharide immunogens, the TF antigen was conjugated to PS B following a similar procedure as noted for synthesizing TF-PS A1 [91].

Sera from TF-PS B immunized mice showed high titers of IgM along with notable amounts of IgG1 and IgG2b isotypes [107]. To further assess the efficacy of our TF-PS B vaccine construct, TF-BSA was synthesized and immunological evaluation revealed a substantial decrease in TF specific antibody recognition elicited by TF-BSA compare to TF-PS B. Antibodies from sera in TF-PS B immunized mice bind selectively to TF expressing MCF-7 carcinoma cells as noted in FACS experiments in high degree. Moreover, anti-TF-PS B antibodies were observed to have a decrease in binding to HCT-116 cells, which are known to express less of the TF antigen [108] as compared to MCF-7 cells. Combining the aforementioned observations, it can be concluded that the TF-PS B immunogen is able to generate TF specific antibody responses.

## 6. Zwitterionic Polysaccharides in Bacterial Vaccines

### 6.1. Sp 1

It has been reported that subcutaneous injections of Sp1 in a mouse model generates a significant amount of Sp1 specific IgG antibody, where induction of the IgG1 isotype is predominant [109]. Due to T-cell activation, Sp1 can be successfully utilized in vaccine preparation to fight against pneumococcal disease caused by pathogens containing this antigen. The first pneumococcal capsular polysaccharide vaccine (PPSV), containing 23 unconjugated capsular polysaccharides, is known to prevent pneumococcal disease caused by 23 different serotypes of *S. pneumonia* (about 88% invasive serotype for pneumococcal disease) and this vaccine is commercialized as PneumoVax^®^23 (Merck, Kenilworth, NJ, USA ). Sp1 is one of the CPSs used in this vaccine [110]. By capitalizing on the immune responses elicited by Sp1, one can predict that incorporation of Sp1 benefits the immunogenicity of the 23 valent vaccine concoction.

### 6.2. CP5 and CP8

CP5 and CP8 have been identified as immunogenic by their ability to activate CD4+ T-cells *in vitro* and in inducing abscess formation in experimental animals [66]. In a study with CP8, it was discovered that it could activate CD4+ T-cells to produce IFN-γ at the site of infection [111]. Evaluation of antisera from attenuated *S. aureus* immunized rabbit revealed that type specific (CP5 and CP8) antibodies can facilitate opsonophagocytosis and bacterial killing by polymorphonuclear cells *in vitro*. Immune responses from these polysaccharides in animal models is not well defined. However, the conjugate of CP5 and CP8 with *Pseudomonas aeruginosa* exotoxin A (Staphvax^TM^) (Nabi Biopharmaceuticals, Rockville, MD, USA) has been observed to induce T-cell mediated immunity [16] towards *S. aureus* infection. Unfortunately Staphvax^TM^ failed in Phase III clinical studies [112]. Therefore, further studies are required to explore the efficacy of these polysaccharides in vaccine preparation before a final verdict can be made.

By capitalizing on the importance of zwitterionic polysaccharides in generating immune responses, attempts have been taken to synthesize the repeating unit of these polysaccharides so that other vaccine strategies can be employed. In this regard, mono- and dimeric repeating units of Sp1 and the synthetic monomeric unit of PS A1 have been reported [113,114]. To date, however, the immunological response from these short oligomers has not shown any promise; most likely that larger fragments will be required for immune activation. Clearly, more creative synthetic routes are necessary to access longer oligomers that will also enable the precise size requirement for studies aimed at completely understanding the size requirement for immunogenicity of these ZPSs.

## 7. Conclusions

This review emphasizes carbohydrate specific immunity induced by entirely carbohydrate-based vaccines highlighting the importance of zwitterionic polysaccharides, isolated from the capsule of commensal anaerobic bacteria, in generating T-cell dependent immune responses. It is well-known that carbohydrates alone are weakly immunogenic, so traditionally covalent attachment to carrier proteins have been generally employed to augment immune responses towards important carbohydrate antigens. In this context, ZPSs have been discussed as an alternative to carrier proteins as these special polysaccharide moieties can be presented to the T-cell receptor as an MHC II complex to generate carbohydrate specific T-cell dependent immunity much like carrier proteins. In addition, ZPSs can bind with toll like receptors, therefore, can play a dual role as both immunogen and adjuvant. This review aimed to detail the advancement of ZPSs-based vaccine design and development that can address many challenges in vaccinology such as targeting cancers and other important diseases. However, MHC I restricted immune responses by ZPS-based vaccines, remain under explored and further understanding is required. MHC I dependent immunity, through carbohydrate-based vaccines, will be a powerful driving force to move vaccines forward in the clinical setting.
